# Relationship between the Mediterranean Diet Score in Pregnancy and the Incidence of Asthma at 4 Years of Age: The Japan Environment and Children’s Study

**DOI:** 10.3390/nu15071772

**Published:** 2023-04-05

**Authors:** Kaita Nakano, Shohei Kuraoka, Masako Oda, Takashi Ohba, Hiroshi Mitsubuchi, Kimitoshi Nakamura, Takahiko Katoh, the Japan Environment and Children’s Study (JECS) Group

**Affiliations:** 1 The South Kyushu Okinawa Unit Center, Faculty of Life Sciences, Kumamoto University, 1-1-1 Honjo, Kumamoto 860-8556, Japan; 2 Department of Pediatrics, Faculty of Life Sciences, Kumamoto University, 1-1-1 Honjo, Kumamoto 860-8556, Japan; 3 Department of Obstetrics and Gynecology, Faculty of Life Sciences, Kumamoto University, 1-1-1 Honjo, Kumamoto 860-8556, Japan; 4 Department of Neonatology, Kumamoto University Hospital, 1-1-1 Honjo, Kumamoto 860-8556, Japan; 5 Department of Public Health, Faculty of Life Sciences, Kumamoto University, 1-1-1 Honjo, Kumamoto 860-8556, Japan

**Keywords:** Mediterranean diet, healthy diet, type 1 allergy, child health

## Abstract

Several scoring methods for the Mediterranean diet, which is considered as a healthy diet, are available, but studies that have compared more than one of these scores are rare. In addition, the applicability of Mediterranean diet scoring has not been sufficiently examined outside of Mediterranean regions. We collected data on the Mediterranean diet during pregnancy and the incidence of type 1 allergies in offspring from the Japan Environment and Children’s Study. Using multiple Mediterranean diet scoring methods, we analyzed the effect of adherence to the Mediterranean diet in pregnancy on the allergies of the offspring. Overall, 46,532 pairs of mothers and children were analyzed. In Japan, a high adherence to the Mediterranean diet during pregnancy was associated with a lower incidence of asthma in the offspring (odds ratio: 0.896, 95% confidence interval: 0.835, 0.962). Furthermore, we found that the selection of the Mediterranean diet scoring method and the setting of the reference value significantly altered the results. Our findings suggest that an appropriate selection of scoring methods and a reference value for food items are important to analyze the effects of adherence to the Mediterranean diet inside and outside of Mediterranean regions.

## 1. Introduction

The Mediterranean diet (MD), which is a traditional diet in Mediterranean coastal areas such as Spain, Italy, and Greece is considered as a healthy diet. The MD is characterized by a high intake of vegetables, fruits, legumes, and cereals, a moderate intake of milk and dairy products, and a low intake of meat and meat products [1]. The Seven Countries Study reported that people in Mediterranean countries had a lower risk of cardiovascular disease, and the MD then became recognized as a healthy dietary style [2]. Furthermore, a meta-analysis of prospective cohort studies showed that adherence to the MD might improve survival in people with a history of cardiovascular disease [3]. A recent report suggested that a higher adherence to the MD during pregnancy might protect against an excess cardiometabolic risk in the offspring [4]. Other studies have shown that mothers with a higher adherence to the MD may have lower perinatal complications such as small for gestational age and gestational diabetes [5,6,7,8]. In addition, a high adherence to the MD during pregnancy might reduce the prevalence of asthma-like symptoms and atopy at 6.5 years old in the offspring [9]. However, several other studies have analyzed the effect of adherence to the MD during pregnancy on children’s allergies, and their effects are still controversial [8,10,11,12,13]. In 2019, a review suggested that a high adherence to the MD during pregnancy and childhood has short-term effects on wheeze in children in early life [11]. However, this finding should be interpreted with caution due to the heterogeneity of the studies. This heterogeneity is due to the existence of multiple MD scores measuring the adherence to the MD. However, studies that have used more than one of these scores and compared them are rare [13,14]. In addition, all of these studies were conducted in Europe and North and South America, so the applicability of MD scoring in pregnant women outside Mediterranean regions has not been confirmed. Therefore, no studies on the relationship between the MD score in pregnant women and allergy in their offspring have been conducted in Japan.

The Japan Environment and Children’s Study (JECS) is a large nationwide birth cohort study that was designed to analyze the effects of various environmental factors on children’s health and development (Appendix A) [15,16]. The JECS enrolled eligible children and their parents and used a self-administered questionnaire to assess the effect of environmental factors. In the present study, we investigated the effect of adherence to the MD during pregnancy on the children’s allergies using data from the JECS. We further examined the applicability of MD scoring to pregnant women using multiple scoring methods.

## 2. Materials and Methods

### 2.1. Study Participants

Approximately 100,000 mother and child pairs were enrolled in the JECS in the first trimester of pregnancy in 15 regional centers in Japan during the recruitment period from January 2011 to March 2014 [15]. Among them, we were able to retrieve all 4 questionnaires in 77,232 pairs. These questionnaires comprised those that were administered at the time of enrollment in the study and at the second/third trimester, a record of the doctor’s examination at delivery, and a health and development questionnaire when the child was 4 years of age. Additionally, pairs that met at least one of the following conditions were excluded: preterm birth (<37 weeks), delivery by cesarean section, pregnancy complications, and smoking during pregnancy. Furthermore, mothers whose energy intake was outside the mean ±2 standard deviation (SD) range were excluded. This information was obtained from the mothers’ daily energy intake from the mid-pregnancy questionnaire data.

### 2.2. Data Collection

All of the surveys used in this study (questionnaire survey during pregnancy, medical record transcript survey at delivery, and questionnaire survey when the child was 4 years of age) were conducted in accordance with the JECS schedule [16]. Maternal dietary surveys were conducted at the time of enrollment in the study and during the second/third trimester. The Food Frequency Questionnaire (FFQ) was distributed to eligible pregnant women, who were asked to respond to this questionnaire [17]. The FFQ has questions regarding the frequency of intake and the portion size in each intake for each food item. We collected the data of dietary habits during mid–late pregnancy from the FFQ conducted in the second/third trimester. We did not adopt the FFQ conducted at the time of enrollment because the questionnaire at the time of enrollment was intended for pre-pregnancy dietary habits. The estimated daily intake (g/day) for food items was calculated for each food item by multiplying the frequency of intake (/day) and the portion size for each intake (g) in the questionnaire. The estimated energy intake each day (kcal/day) for each food item was then calculated by multiplying that intake by the energy in each intake (kcal/g). These data were extracted from the jecs-ta-20190930 dataset. Information on the number of weeks of gestation at the time of delivery, method of delivery, and presence or absence of pregnancy complications was collected from the doctor’s examination record at the time of delivery. These data were extracted from the jecs-ta-20190930 dataset. The smoking history was collected at the time of enrollment and in the second/third trimester from the jecs-ta-20190930 dataset. Information of five allergic diseases (asthma, food allergy, atopic dermatitis, allergic conjunctivitis, and allergic rhinitis) in children aged 4 years was collected by the parents in response to a questionnaire. The questionnaire was a modified version of the International Study of Asthma and Allergies in Childhood questionnaires for 6–7 years old, which was translated and validated in Japanese [18,19,20]. These data were extracted from the jecs-qa-20210401 dataset.

### 2.3. Calculation of the MD Score

The MD scores were calculated using indices of the Mediterranean diet scale (MDS) [21], the relative Mediterranean diet (rMED) [22], and the Mediterranean diet scale in pregnancy (PMDS) [9].

In the MDS, vegetables, legumes, fruits, nuts, cereals, and fish are categorized as beneficial components of the MD, while meat and dairy products are classified as detrimental components. A score of 0 or 1 point is assigned on the basis of each median intake of beneficial components or detrimental components, respectively. Alcohol intake and fat intake are scored on the basis of presumed beneficial standards and the ratio of monounsaturated to saturated lipids, respectively. The range of the final score of the MDS is from 0 to 9 points. The details of this scoring have previously been described [21].

In the rMED, we used the same components as those in the MDS. A score of 0, 1, or 2 points was assigned on the basis of each tertile intake. In addition, the fat intake was evaluated only from the olive oil intake. The range of the final score of the rMED was from 0 to 18 points. The details of scoring of the rMED were as previously described [22].

The PMDS is a scoring method that was modified from the MDS for pregnant women [9]. Unlike the MDS, dairy products are categorized as a beneficial component in the PMDS. In addition, alcohol and fat ratios are excluded in this method. The range of the final score of the PMDS is from 0 to 7 points. The details of the PMDS are as described previously [9].

Each reference value of these scoring methods was obtained from each researched population living in the Mediterranean region, as described previously [9,21,22]. To evaluate adherence to the Mediterranean diet as an absolute value, we selected the reference value obtained from the Mediterranean population in previous reports for scoring in the current study. Additionally, we performed another analysis using the reference value obtained from the JECS participants as a comparison with the analysis using the reference value obtained from the Mediterranean population in previous reports.

### 2.4. Main Outcome

In this study, the incidence of diseases classified as type 1 allergies, which are caused by immunoglobulin E antibodies produced in response to environmental factors, was adopted as the outcome [23,24,25]. Parents filled out the JECS questionnaire when their children were aged 4 years to determine if their child had each of the five following typical type 1 allergies: asthma, food allergy, atopic dermatitis, allergic conjunctivitis, and allergic rhinitis.

### 2.5. Statistical Analysis

Data are presented as the mean with standard deviation (SD) or 95% confidence interval (CI). In this study, we conducted a statistical analysis of the effect of a high adherence to the MD in the second/third trimester on the incidence of each type 1 allergic disease in the mothers’ offspring at 4 years of age using the Pearson’s chi-square test with the Yates continuity correction. Differences of *p* value < 0.05 were considered statistically significant. All statistical analyses were performed with EZR (Version 1.55; Saitama Medical Center, Jichi Medical University, Saitama, Japan), which is a graphical user interface for R (The R Foundation for Statistical Computing, Vienna, Austria). More precisely, EZR is a modified version of R commander designed to add statistical functions frequently used in biostatistics [26].

## 3. Results

### 3.1. Participant Selection

**Figure 1:**
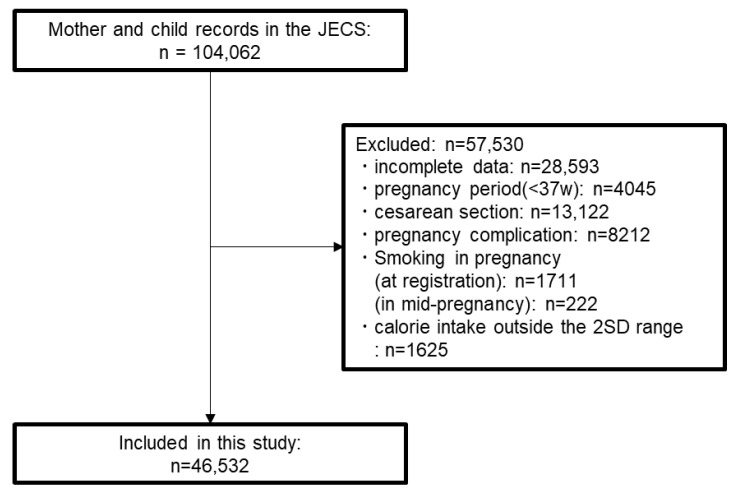
Flow diagram of the participants. From 104,062 records of mothers and children in the JECS, we excluded pairs who met the exclusion criteria (preterm delivery, cesarean section, any pregnancy complications, smoking during pregnancy, and outside of the mean ± 2 SD range of the energy intake from the mid-pregnancy FFQ).

From a total of 104,062 pairs of participants in the JECS, we excluded those who did not submit the questionnaire, and 77,232 pairs remained. After excluding 1763 pairs with incomplete data, we excluded 27,312 pairs that met any of the following conditions: preterm delivery (pregnancy period < 37 weeks), cesarean section, any pregnancy complications, and smoking during pregnancy. The mean energy intake of the remaining 48,157 pairs that were obtained from the mid-pregnancy FFQ was approximately 1732 kcal/day. We excluded mothers whose energy intake was less than the mean −2 SDs (263.3 kcal/day) or higher than the mean +2 SDs (3201.6 kcal/day). Finally, we included 46,532 pairs of mothers and children among all participants in the JECS (Figure 1).

### 3.2. Baseline Characteristics

The characteristics of the participants are shown in Table 1. Among the mothers, almost half had one or more allergic diseases. The prevalence of each disease was 9.3% for asthma, 4.4% for food allergy, 15.9% for atopic dermatitis, 10.0% for allergic conjunctivitis, and 36.4% for allergic rhinitis. These values were almost equal to the prevalence rates in the whole population of the JECS [27]. In children, the prevalence rates were 8.2% for asthma, 5.6% for food allergy, 8.5% for atopic dermatitis, 2.6% for allergic conjunctivitis, and 7.6% for allergic rhinitis.

### 3.3. Main Results

#### 3.3.1. MDS Analysis

We first examined the MDS, which is the most popular scoring method (Table 2). The mean (±SD) value of the MDS was 4.05 ± 0.83 points. Similar to a previous study, we divided mothers into the MDS high group (≥5 points) and the MDS low group (≤4 points) [21]. As a result, we grouped 13,336 pairs in the high group and 33,196 pairs in the low group in the MDS analysis. No significant differences in allergies were found between the two groups. The high and low groups were equally affected by all allergic diseases, where 23.9% children in the high group and 24.4% in the low group had one or more allergies. We also divided all participants (*n* = 46,532) into two groups, which comprised one with a maternal allergy (*n* = 23,176) and the other without a maternal allergy (*n* = 23,356). No significant differences in allergies were observed between the high and low groups with and without a maternal allergy.

#### 3.3.2. rMED Analysis

We examined the rMED, which was modified from the MDS (Table 3). The mean (±SD) value of the rMED was 8.02 ± 1.93. Similar to a previous study, we divided mothers into the rMED high group (≥11 points) and the rMED low group (≤10 points) [22]. As a result, we grouped 4476 pairs in the high group and 42,056 pairs in the low group in the rMED analysis. No significant differences in allergies were found between the two groups. The high and low groups were equally affected by all five allergic diseases, where 25.0% of children in the high group children and 24.2% in the low group had one or more allergies. We also analyzed participants with and without a maternal allergy. In the condition with maternal allergies, the rMED high group showed a higher incidence of food allergy than the rMED low group (*p* value: 0.043). However, this difference was not observed in the condition without maternal allergies. Therefore, this rMED analysis suggests that addressing the association between the mother’s MD adherence and the offspring’s allergies is difficult.

#### 3.3.3. PMDS Analysis

We examined the PMDS, which was modified from the MDS (Table 4). The mean (±SD) value of the PMDS was 3.93 ± 0.87. Similar to a previous study, we divided the mothers into the PMDS high group (≥4 points) and the PMDS low group (≤3 points) [9]. As a result, we grouped 31,920 pairs in the high group and 14,612 pairs in the low group in the PMDS analysis. The high group had a significantly lower rate of one or more allergies than the low group (*p* value: 0.020). The incidence of asthma was significantly lower in the high group than in the low group (odds ratio: 0.896, 95% CI: 0.835, 0.962). No significant difference in the incidence of the other diseases, except for asthma, was found between the two groups. We also analyzed the condition with and without a maternal allergy. In the group with a maternal allergy, no significant difference in the incidence rates for all five diseases was found between the high and low groups. In the group without a maternal allergy, the incidence of asthma was significantly lower in the high group than in the low group (odds ratio: 0.827, 95% CI 0.740, 0.925). No significant difference in the incidence of other diseases was observed between the high and low groups. Additionally, in the group without a maternal allergy, the number of patients with at least one of the five allergic diseases was significantly lower in the high group than in the low group (*p* value: 0.004). Although a previous study reported that the incidence of atopic dermatitis was lower in the high group than in the low group, in the present study, the incidence of dermatitis was not significantly different between these groups [9].

Although the present analysis targeted 46,532 participants in whom factors affecting the incidence of allergy were excluded, similar results were obtained when the analysis was performed in 74,487 participants before excluding these factors (Table S1).

In addition, our inclusion criteria included mothers with a dietary intake of <1000 kcal/day. To determine the effect of this low dietary intake on the results of the PMDS analysis, we also examined the incidence of asthma in each dietary intake range. We found that the PMDS high group had a lower incidence of asthma in all dietary intake ranges than the PMDS low group (Figure S1).

The analyses above-mentioned were performed using reference values that were median values obtained from a population living in the Mediterranean region described in a previous report [22]. We also performed the same analysis using the reference value obtained from the JECS participants living in Japan. Table 5 shows the comparison of the median values in this previous study and those in the current study. The median consumption (g/day) of vegetables, fruits/nuts, and meat in the current study was lower than that in the previous report. However, the median consumption of legumes and cereals was higher in the current study than in the previous report. We then performed a PMDS analysis using these median values in the JECS (Table 6). The number of patients with one or more of the five allergic diseases in this analysis was higher in the high group than in the low group (*p* value: 0.012), but the incidence of asthma was not different between the high and low groups while the high group had a higher incidence of atopic dermatitis. These findings suggest that changing the reference value can significantly alter the results of the PMDS analysis.

## 4. Discussion

Various scoring methods have been used in previous studies to assess the MD, and the most extended and frequently used score is the MDS [8,10,11,12,13]. Recently, some scoring methods modified from the MDS have also been used [9,13,22]. Among these scoring methods, the rMED has widened the range of final scores from 9 full points to 18 full points, allowing a more detailed distribution of adherence to the MD [22]. In addition, the PMDS was adapted to pregnant women by modifying the scoring criteria according to their characteristics. The PMDS was used in a study on pregnant women’s diets and allergies in children [9]. Therefore, in this study, we selected the MDS, rMED, and PMDS to analyze whether a high adherence to the MD during pregnancy affects the incidence of type 1 allergic diseases at 4 years of age.

Our study showed that the incidence of asthma was significantly lower in the high group than in the low group using the PMDS. However, no significant differences in allergic diseases were found in the other two scoring methods (MDS and rMED) between the high and low groups. The main differences between the PMDS and MDS or rMED are the classification of dairy products and the presence of alcohol items. Both of these changes were taken into account for the characteristics of pregnant women. By including dairy products as a beneficial component, a population of approximately 20,000 individuals, who were in the low group by a slight margin in the MDS analysis, were admitted to the high group in the PMDS analysis. This change in the cutoff line may have allowed for an accurate assessment of the impact of the Mediterranean diet. Therefore, a scoring method that is designed by considering pregnant women’s characteristics would be more appropriate for MD studies involving pregnant women. However, which scoring method should be used in each case has not been clarified, and careful consideration is required. In addition, we used the Mediterranean diet scoring with cutoffs in the present study as previously described. Further research is needed to analyze continuous outcomes such as whether a higher Mediterranean diet scoring enhances the effect.

The participants in this study were residents of Japan, and their dietary habits are likely to be different from those in the Mediterranean region (Table 5). In particular, only 0.4% of the participants in this study were scored on the olive oil intake section in the rMED analysis. We also performed an analysis using the median values of the current participants in the JECS, and did not find a positive effect of high adherence to the MD on the incidence of asthma in the offspring. This finding suggests that selecting absolute measures based on dietary habits in the Mediterranean region, rather than a relative measure, may be preferable for calculating a reliable MD score.

This study had several limitations. The primary outcome was the incidence of allergy at 4 years of age, and allergies before 3 years of age were not considered. We used data from a questionnaire that was administered during the second or third trimester for the mother’s diet and did not take into account the effect of the pre-pregnancy diet or the postnatal diet of the infants. Although other factors such as passive smoking, breastfeeding, and the family’s economic status may affect the child’s incidence of allergies, we could not fully consider these conditions in this study [28,29,30]. A detailed analysis that includes such biases is required to determine the appropriate relationship between adherence to the MD and allergies. In addition, all questionnaires for mothers and children were self-administered and may have been prone to the subjectivity of the person filling them out. In particular, the incidence of atopy was also self-determined and not based on a diagnosis by doctors. This unreliability may be one of the reasons why we could not find a relationship between adherence to the MD and atopy.

The mechanisms of how adherence to the MD in pregnancy affects the incidence of asthma in offspring have not yet been determined. Recently, several reports have been conducted on the mechanisms of the relationship between MD and allergies. Gut bacteria have been the focus as an important factor involved in these mechanisms. Higher MD scores have been found to increase the gut microbial diversity and short-chain fatty acid-producing bacteria [31]. Additionally, the administration of the probiotic *Lactobacillus* improves and prevents symptoms of allergic diseases [32]. Furthermore, the gut microbiota at birth in neonates born by vaginal delivery is rich in bacteria derived from the mother’s gut microbiota [33]. Maternal adherence to the MD may increase the mother’s gut microbial diversity and affect the incidence of asthma in the offspring by changing the gut microbiota of the offspring.

## 5. Conclusions

Our study, using multiple scoring methods of the MD, showed that the PMDS score during pregnancy is associated with the incidence of asthma in children at 4 years of age. This result may shed light on the applicability of MD scoring outside Mediterranean regions. Furthermore, this study suggests that the appropriate selection of MD scoring methods and a reference value for food items are important for analyzing the effects of adherence to the MD.

## Figures and Tables

**Table 1:** Profile of the participants.

Type1 Allergy	Mothers (*n* = 46,532)	Children (*n* = 46,532)
One or more (%)	23,176 (49.8)	11,293 (24.3)
Asthma (%)	4324 (9.3)	3815 (8.2)
Food allergy (%)	2050 (4.4)	2616 (5.6)
Atopic dermatitis (%)	7394 (15.9)	3954 (8.5)
Allergic conjunctivitis (%)	4661 (10.0)	1223 (2.6)
Allergic rhinitis (%)	16,930 (36.4)	3526 (7.6)

**Table 2:** Comparison of allergic outcomes between the two MDS groups.

	All Children			Maternal Allergies Group			No Maternal Allergies Group		
Type1 Allergy	MDS High (*n* = 13,336)	MDS Low (*n* = 33,196)	*p*	MDS High (*n* = 6634)	MDS Low (*n* = 16,542)	*p*	MDS High (*n* = 6702)	MDS Low (*n* = 16,654)	*p*
One or more (%)	3182 (23.9)	8111 (24.4)	0.196	1941 (29.3)	4933 (29.8)	0.406	1241 (18.5)	3178 (19.1)	0.327
Asthma (%)	1060 (7.9)	2755 (8.3)	0.219	641 (9.7)	1706 (10.3)	0.144	419 (6.3)	1049 (6.3)	0.917
Food allergy (%)	747 (5.6)	1869 (5.6)	0.921	449 (6.8)	1132 (6.8)	0.860	298 (4.4)	737 (4.4)	0.972
Atopic dermatitis (%)	1157 (8.7)	2797 (8.4)	0.392	729 (11.0)	1719 (10.4)	0.189	428 (6.4)	1078 (6.5)	0.830
Allergic conjunctivitis (%)	358 (2.7)	865 (2.6)	0.654	246 (3.7)	578 (3.5)	0.450	112 (1.7)	287 (1.7)	0.824
Allergic rhinitis (%)	981 (7.4)	2545 (7.7)	0.260	648 (9.8)	1662 (10.0)	0.537	333 (5.0)	883 (5.3)	0.315

MDS: Mediterranean diet scale.

**Table 3:** Comparison of allergic outcomes between the two rMED groups.

	All Children			Maternal Allergies Group			No Maternal Allergies Group		
Type1 Allergy	rMED High (*n* = 4476)	rMED Low (*n* = 42,056)	*p*	rMED High (*n* = 2265)	rMED Low (*n* = 20,911)	*p*	rMED High (*n* = 2211)	rMED Low (*n* = 21,145)	*p*
One or more (%)	1117 (25.0)	10,176 (24.2)	0.268	704 (31.1)	6170 (29.5)	0.125	413 (18.7)	4006 (18.9)	0.783
Asthma (%)	364 (8.1)	3451 (8.2)	0.887	222 (9.8)	2125 (10.2)	0.614	142 (6.4)	1326 (6.3)	0.816
Food allergy (%)	266 (5.9)	2350 (5.6)	0.344	178 (7.9)	1403 (6.7)	0.043	88 (4.0)	947 (4.5)	0.303
Atopic dermatitis (%)	390 (8.7)	3564 (8.5)	0.606	253 (11.2)	2195 (10.5)	0.340	137 (6.2)	1369 (6.5)	0.645
Allergic conjunctivitis (%)	131 (2.9)	1092 (2.6)	0.206	96 (4.2)	728 (3.5)	0.074	35 (1.6)	364 (1.7)	0.695
Allergic rhinitis (%)	354 (7.9)	3172 (7.5)	0.395	249 (11.0)	2061 (9.9)	0.093	105 (4.7)	1111 (5.3)	0.333

rMED: relative Mediterranean diet.

**Table 4:** Comparison of allergic outcomes between the two PMDS groups.

	All Children			Maternal Allergies Group			No Maternal Allergies Group		
Type1 Allergy	PMDS High (*n* = 31,920)	PMDS Low (*n* = 14,612)	*p*	PMDS High (*n* = 16,109)	PMDS Low (*n* = 7067)	*p*	PMDS High (*n* = 15,811)	PMDS Low (*n* = 7545)	*p*
One or more (%)	7646 (24.0)	3647 (25.0)	0.020	4735 (29.4)	2139 (30.3)	0.185	2911 (18.4)	1508 (20.0)	0.004
Asthma (%)	2533 (7.9)	1282 (8.8)	0.002	1598 (9.9)	749 (10.6)	0.120	935 (5.9)	533 (7.1)	0.0008
Food allergy (%)	1772 (5.6)	844 (5.8)	0.340	1080 (6.7)	501 (7.1)	0.297	692 (4.4)	343 (4.5)	0.579
Atopic dermatitis (%)	2687 (8.4)	1267 (8.7)	0.373	1695 (10.5)	753 (10.7)	0.779	992 (6.3)	514 (6.8)	0.124
Allergic conjunctivitis (%)	844 (2.6)	379 (2.6)	0.776	581 (3.6)	243 (3.4)	0.550	263 (1.7)	136 (1.8)	0.476
Allergic rhinitis (%)	2393 (7.5)	1133 (7.8)	0.340	1600 (9.9)	710 (10.0)	0.807	793 (5.0)	423 (5.6)	0.062

PMDS: Mediterranean diet scale in pregnancy.

**Table 5:** Comparison of the median values used in a previous study with those in the current study.

	Median [g/Day]	
Food Item	Previous Study	Current Study
Vegetable	499.6	158.0
Legume	6.7	52.1
Fruit and nut	356.3	106.8
Cereal	139.7	431.5
Fish	18.8	27.1
Dairy product	191.1	172.1
Meat	89.8	57.4

**Table 6:** Comparison of the allergic outcomes between the two PMDSnM groups.

	All Children		
Type 1 Allergy	PMDSnM High (*n* = 23,149)	PMDSnM Low (*n* = 23,383)	*p*
One or more (%)	5735 (24.8)	5558 (23.8)	0.012
Asthma (%)	1926 (8.3)	1889 (8.1)	0.351
Food allergy (%)	1303 (5.6)	1313 (5.6)	0.965
Atopic dermatitis (%)	2032 (8.8)	1922 (8.2)	0.032
Allergic conjunctivitis (%)	638 (2.8)	585 (2.5)	0.092
Allergic rhinitis (%)	1806 (7.8)	1720 (7.4)	0.072

PMDSnM: Mediterranean diet scale in pregnancy using the median values in non-Mediterranean countries.

## Data Availability

Data are unsuitable for public deposition due to ethical restrictions and the legal framework of Japan. The Act on the Protection of Personal Information (Act No. 57 of 30 May 2003, amendment on 9 September 2015) prohibits publicly depositing data containing personal information. The Ethical Guidelines for Medical and Health Research Involving Human Subjects enforced by the Japan Ministry of Education, Culture, Sports, Science and Technology, and the Ministry of Health, Labor and Welfare also restrict the open sharing of epidemiological data. All inquiries about access to data should be sent to the following address: jecs-en@nies.go.jp. The person responsible for handling enquiries sent to this email address is Shoji F. Nakayama, JECS Program Office, National Institute for Environmental Studies.

## Supplementary Materials

The following supporting information can be downloaded at: https://doi.org/10.5281/zenodo.7584187 (accessed on 2 April 2023), Table S1: Comparison of allergic outcomes between the two PMDS groups before exclusion; Figure S1: Incidence of asthma in each dietary intake range.

## Appendix A

Members of the JECS Group as of 2022: Michihiro Kamijima (Principal Investigator, Nagoya City University, Nagoya, Japan), Shin Yamazaki (National Institute for Environmental Studies, Tsukuba, Japan), Yukihiro Ohya (National Center for Child Health and Development, Tokyo, Japan), Reiko Kishi (Hokkaido University, Sapporo, Japan), Nobuo Yaegashi (Tohoku University, Sendai, Japan), Koichi Hashimoto (Fukushima Medical University, Fukushima, Japan), Chisato Mori (Chiba University, Chiba, Japan), Shuichi Ito (Yokohama City University, Yokohama, Japan), Zentaro Yamagata (University of Yamanashi, Chuo, Japan), Hidekuni Inadera (University of Toyama, Toyama, Japan), Takeo Nakayama (Kyoto University, Kyoto, Japan), Tomotaka Sobue (Osaka University, Suita, Japan), Masayuki Shima (Hyogo Medical University, Nishinomiya, Japan), Hiroshige Nakamura (Tottori University, Yonago, Japan), Narufumi Suganuma (Kochi University, Nankoku, Japan), Koichi Kusuhara (University of Occupational and Environmental Health, Kitakyushu, Japan), and Takahiko Katoh (Kumamoto University, Kumamoto, Japan).
